# Whole-genome sequence of *Streptomyces* sp. F41, a potent insecticidal *Streptomyces* isolated from soil in Sichuan, China

**DOI:** 10.1128/mra.00306-24

**Published:** 2024-06-06

**Authors:** Shige Du, Cheng Guo, Junhong Wei, Chunfeng Li, Zeyang Zhou

**Affiliations:** 1 State Key Laboratory of Resource Insects, Southwest University, Chongqing, China; 2 Chongqing Key Laboratory of Microsporidia Infection and Prevention, Southwest University, Chongqing, China; 3 College of Life Sciences, Chongqing Normal University, Chongqing, China; Montana State University, Bozeman, Montana, USA

**Keywords:** actinomycetes, *Streptomyces*, genome analysis

## Abstract

*Streptomyces* sp. F41 is a potent insecticidal metabolite producing actinomycetes isolated from the topsoil, and the complete genome sequence was determined. The genome consists of 8,343,496 bp, with 7,221 genes and a GC content of 71.84%.

## ANNOUNCEMENT

Between 20% and 40% of global crop production loss is caused by pests; chemical insecticides are most widely used to protect crop plants from harmful insects. Meanwhile, extensive use of chemical pesticides brings negative impact on the environment, humans, and non-target organisms (1). For these reasons, there is an urgent need for developing environmentally friendly biological approaches for pest management.

Pest management by biological means is effective and an eco-friendly strategy; bio-insecticidal metabolites isolated from microorganisms have been gaining increased attention and interest (2). Actinomycetes represent one of the most important and widely studied microbial resources in pest management because they produce various forms of active compounds for use as pesticides (3). Here, we announce the complete genome sequence of *Streptomyces* sp. F41, a soil-dwelling actinomycetes with anti-insect potential.

*Streptomyces* sp. F41 was isolated from topsoil samples collected from Kangding, Sichuan, China (29.913658 N, 102.000935 E). For bacteria isolation, 50 µg soil sample was resuspended by 1 mL sterilized saline water and followed by 10-fold serial dilution to 10^−8^. One hundred microliters of each dilution is added to Gauze’s synthetic medium no. 1 and incubated at 28°C for 5 days (4). Strain F41 was isolated by cultivating a single colony upon Mannitol Soya Flour medium (5) for 5 days at 28°C to achieve an axenic culture; three iterations of subculturing were conducted from a single colony.

Spores of strain F41 were harvested by 1 mL sterilized saline water and inoculated in Luria-Bertani liquid medium for 2 days at 30°C to provide mycelium for DNA extraction (6). TGuide S96 Magnetic Soil/Stool DNA Kit (TIANGEN, China) was used for DNA extraction. G-tube (Covaris, USA) was used for DNA shearing, and size selection was performed with Agilent Technologies 2100. The total DNA was then subjected to whole-genome sequencing at BioMarker Technologies Co. Ltd. (Beijing, China). The SMRTbell express template prep kit 2.0 and PacBio Sequel II system were used to create a DNA library and sequence the genome, respectively (7). PacBio CCS sequencing mode generated 27,020 raw reads, the N_50_ of raw reads was 9,874 bp; reads with length shorter than 2 kb were removed to get 26,993 filtered subreads. The filtered subreads were then assembled by Hifiasm (8). Coding gene prediction was processed by NCBI Prokaryotic Genome Annotation Pipeline v.6.7(9, 10). Default parameters were used, except where otherwise noted.

The assembled genome was 8,343,496 bp in length with two contigs: contig 1 (8,310,873 bp) and contig 2 (32,623 bp) with a GC content of 71.86% and 68.54%, respectively. The coverages of the contig 1 and contig 2 are 28.6× and 62.3×, respectively. As no overlapping sequence was found at ends of the assembly sequences, both contigs are linear. The genome annotation identified 7,221 genes from both contigs, consisting of 7,024 coding sequences, 111 pseudo genes, 68 tRNAs, and 18 rRNAs.

## Data Availability

The complete genome of *Streptomyces* sp. F41 is available at NCBI GenBank under the accession number CP149796-CP149797. The BioSample number is SAMN40537137 and BioProject number is PRJNA1089331. The Raw data reads are available in the Sequence Read Archive (SRA) under the accession number SRR28393140.
